# The relationship between climate change, globalization and non-communicable diseases in Africa: A systematic review

**DOI:** 10.1371/journal.pone.0297393

**Published:** 2024-02-23

**Authors:** Alhassan Siiba, Joseph Kangmennaang, Leonard Baatiema, Isaac Luginaah

**Affiliations:** 1 School of Kinesiology and Health Studies, Queen’s University, Kingston, Ontario, Canada; 2 Department of Health Policy, Planning and Management, School of Public Health, University of Ghana Legon, Greater Accra Region, Ghana; 3 Department of Global Health and Population, Harvard T.H. Chan School of Public Health, Boston, Massachusetts, United States of America; 4 Oxford Centre for Global Health Research, Nuffield Department of Medicine, University of Oxford, Oxford, United Kingdom; 5 Department of Geography and Environment, University of Western Ontario, Ontario, Canada; MRC Unit The Gambia at LSHTM, GAMBIA

## Abstract

Climate change and non-communicable diseases (NCDs) are considered the 21^st^ Century’s major health and development challenges. Both pose a disproportionate burden on low- and middle-income countries that are unprepared to cope with their synergistic effects. These two challenges pose risks for achieving many of the sustainable development goals (SDGs) and are both impacted by globalization through different pathways. While there are important insights on how climate change and or globalization impact NCDs in the general literature, comprehensive research that explores the influence of climate change and or globalization on NCDs is limited, particularly in the context of Africa. This review documents the pathways through which climate change and or globalization influence NCDs in Africa. We conducted a comprehensive literature search in eight electronic databases—Web of Science, PubMed, Scopus, Global Health Library, Science Direct, Medline, ProQuest, and Google Scholar. A total of 13864 studies were identified. Studies that were identified from more than one of the databases were automatically removed as duplicates (n = 9649). Following the Preferred Reporting Items for Systematic Reviews and Meta-Analyses (PRISMA) guidelines, a total of 27 studies were eventually included in the final review. We found that the impacts of climate change and or globalization on NCDs act through three potential pathways: reduction in food production and nutrition, urbanization and transformation of food systems. Our review contributes to the existing literature by providing insights into the impact of climate change and or globalization on human health. We believe that our findings will help enlighten policy makers working on these pathways to facilitate the development of effective policy and public health interventions to mitigate the effects of climate change and globalization on the rising burden of NCDs and goal 3 of the SDG, in particular.

## 1. Introduction

Climate change and non-communicable diseases (NCDs) are considered the 21^st^ century’s major health and development challenge [1–5]. Both pose a disproportionate burden on low- and middle-income countries (LMICs) that are unprepared to cope with their synergistic effects. These challenges threaten the achievement of many of the sustainable development goals (SDGs), especially goals 1, 2 and 3, which are related to reducing the prevalence of NCDs and managing existing NCD conditions [6]. While NCDs are diverse, the four most common ones are cardiovascular disease (CVDs), including heart attack and stroke; cancers; chronic respiratory disease (chronic obstructive pulmonary disease and asthma); and diabetes [7]. NCDs are responsible for much of the world’s disease burden, accounting, approximately, for 71% of all global deaths, which has significant economic and social implications [8]. The World Health Organization (WHO), for instance, has projected that surveillance and management of NCDs could cost the global economy up to US$47 trillion by 2030 [9]. Other studies have also suggested that NCDs are the leading cause of medical impoverishment, disproportionately clustered in the LMICs [10, 11].

There is a marked intersection between globalization and climate change in relation to health, which necessitates a comprehensive understanding of their individual and or synergistic impacts on NCDs. For instance, globalization, notably through increased industrialization and global trade, has been implicated in the heightened frequency and severity of climate change events, especially through the emission of greenhouse gases [12–15]. Such climate change events, like floods and droughts, have adversely affected agricultural practices, compromised food security, and inducted population displacements, thereby exposing affected individuals to communicable diseases and diet-related NCDs [12, 16]. Conversely, climate change events have also influenced the temporal and spatial dimensions of globalization, particularly in the context of food demand, and the evolving landscape of global supply chains [17]. Specifically, extreme climate events, which have led to decreased availability of locally cultivated foods, have prompted the globalization of high-energy dense foods under the pretext of providing food aid to affected populations [18]. This has consequently impacted the food system, including changes in dietary behaviors that are detrimental to human health [18–21].

Climate change and globalization are inherently tied to the increasing risk factors and prevalence of NCDs [22]. For instance, the tendency for rising temperatures to increase the incidence of CVDs among populations with pre-existing health conditions has been well documented [23]. Eze and colleagues, in their systematic review and meta-analyses of the association between ambient air pollution and diabetes mellitus in the context of Europe and North America, have argued that ambient air pollution can directly influence the prevalence of NCDs [24]. Allen-Scott et al. [25] also argued that while globalization has led to increased access to medical technologies, it has also indirectly contributed to unhealthy lifestyle changes and behaviors that have increased and still have the potential to increase the risks for NCDs. This argument has been collaborated by the World Health Organization (WHO). Essentially, the WHO has acknowledged that the globalization of unhealthy lifestyles has increased the burden of NCDs worldwide particularly due to the spread and consumption of processed foods and increased sedentary lifestyles [5].

Increasing frequency and severity of climate change events can worsen the already weakened health systems and healthcare delivery in Africa, with potential direct and or indirect consequences on NCD conditions [26]). Such circumstances may be intense within the context of the increasing health burden of communicable diseases such as HIV/AIDS and COVID-19 [20, 27] and the unpredictable reoccurrences of Ebola outbreaks in the African continent [28]. Severe drought incidences are often related to human migration and displacement and have also been linked to the spread of infectious diseases to new areas [29]. For instance, during the Mozambican drought of 1991–1992, an estimated 1.3 million people were forced to seek refuge in urban areas of neighboring countries [30]. Zimbabwe also experienced an outbreak of cholera after an influx of these refugees from Mozambique, building up large refugee camps with limited resources to promote good health [31].

While the above discussions give some important insights about how climate change and globalization have and could directly or indirectly impact NCDs, comprehensive research that explores how they individually or jointly impact NCDs is limited in the context of Africa. Thus, our aim in this review is to contribute to the existing literature by providing additional insights into the impact of climate change and or globalization on human health, specifically NCDs. Findings from this review will contribute to the discussions on climate change, globalization, and health, and has the potential to inform the development of effective policy and public health interventions aimed at mitigating the effects of climate change and or globalization as drivers of NCD burden in Africa. The review is presented in six sections, starting with the introduction; section 2 covers the methods used in the review, including the search for relevant literature and the criteria for inclusion and exclusion. Section 3 presents the data abstraction and analysis/synthesis process. Section 4 presents the results from the review, highlighting the general characteristics of the included literature, and discusses the major pathways that emerged from the review. Section 5 discusses the key findings, while the limitations and conclusion are presented in Section 6.

## 2. Methods

### 2.1 Search strategy

**Fig 1:**
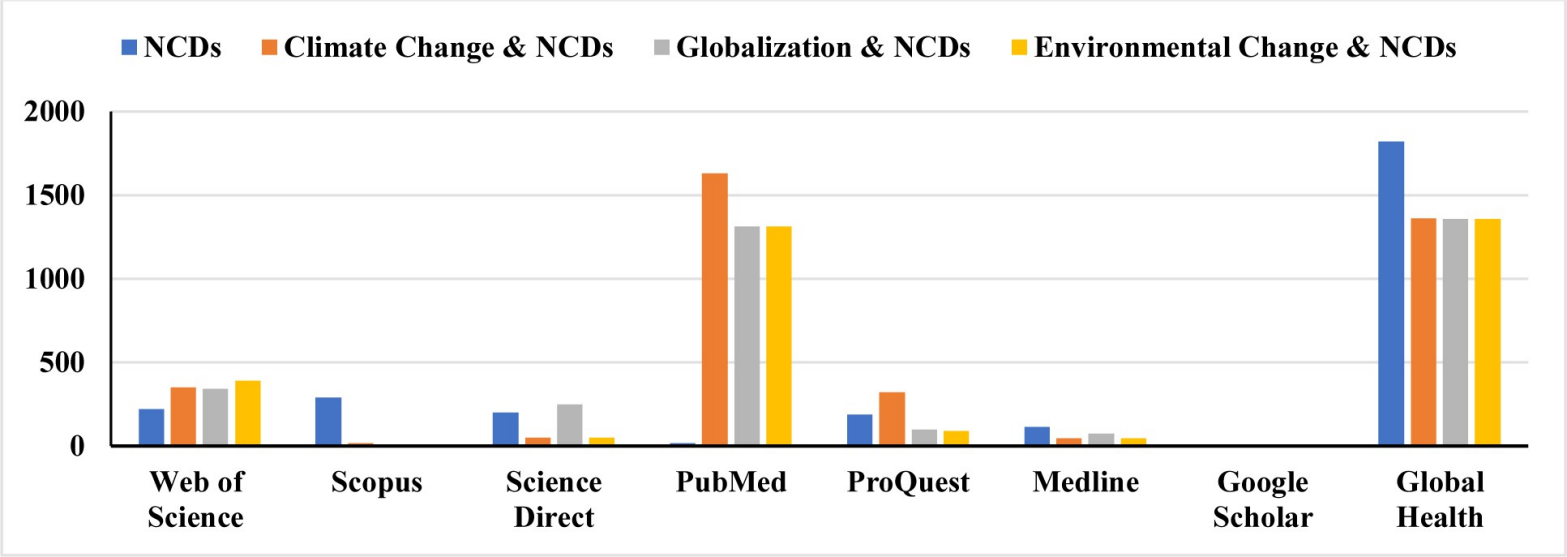
Number of studies identified from the search categories.

We conducted a comprehensive literature search in accordance with the Preferred Reporting Items for Systematic Reviews and Meta-Analyses (PRISMA) [32]. The literature searches were conducted in eight electronic databases—Web of Science, PubMed, Scopus, Global Health Library, Science Direct, Medline, ProQuest, and Google Scholar. We also conducted reference checking in some of the studies retrieved from these databases for relevant additional studies. The most recent search for potential studies from the above databases was conducted on 2^nd^ April, 2023. For the main search, a Boolean search, using a combination of “AND” and “OR”, was utilized in the search process. The first author (AS) conducted the search using the search terms “non-communicable diseases”, “noncommunicable diseases”, “climate change”, “NCDs”, “globalization” and “environmental change”. The search was grouped into four categories—NCDs, Climate Change and NCDs, Globalization and NCDs, and Environmental Change and NCDs (see **S1 Table**)—and the number of studies identified from each search category was recorded, as shown in **Fig 1**.

### 2.2 Eligibility criteria

#### 2.2.1 Type of studies and context

Our electronic searches were limited to peer-reviewed and original research conducted in English. The inclusion criteria required the studies to be focused on either climate change, globalization or environmental change or a combination of them and their relationship with non-communicable diseases (NCDs) in the context of Africa. We excluded non-English studies as the authors do not speak or understand these languages and do not want to misinterpret or misrepresent these studies. Systematic review papers as well as studies that were conducted outside the African continent were excluded. However, where a review was identified, the eligible studies in the review was extracted and included individually. No restrictions were put on the year of publication of the studies or studies published in any specific peer-reviewed journal.

#### 2.2.2 Population and outcome of interest

We considered all population groups, as were discussed in the original studies. The outcomes of interest included the direct or indirect influence of climate change or globalization on NCDs in Africa. In this regard, and to help ensure that the systematic review process would be manageable in scope, a decision was made to limit the studies to only those that demonstrated evidence of relationship between ‘climate change and NCDs’ or ‘globalization and NCDs’, or a combination of globalization, climate/environmental change and NCDs and the measured dependent variables of the studies. Thus, any studies that discussed the real or the potential influence of climate change or globalization on the risk factors for NCDs (e.g., physical inactivity, increased smoking, and alcohol intake, etc.) were included in the final review.

### 2.3 Study selection

Selection of the eligible studies was conducted following a four-step process. Firstly, a single reviewer (AS) screened and imported the full citations of the identified studies into Covidence. Secondly, titles and the abstracts of the imported studies were screened by two reviewers (AS and JK) to determine if such studies should be included based on the inclusion criteria established for this review. The third stage involved cross-checking of the eligible studies for full-text review. This stage was conducted by a third reviewer to help minimize potential selection and inclusion bias by the first two reviewers. The fourth stage involved full-text screening to select studies meeting the inclusion criteria. Two reviewers (AS and JK) screened the studies and a cross-checked done by a third reviewer (LB) as an additional measure to reduce inclusion bias in the final step [33]. Using a standardized pre-extraction form, all the eligible studies were extracted. The extracted studies were imported into EndNote (v21) for the final review.

### 2.4 Data extraction

A standardized evidence table was developed and trialed with the first 2–3 articles to ensure it is robust enough to comprehensively capture all relevant data to answer the review questions. Data about the author(s), year of publication of the study, the specific country in which the study was conducted, the focus of the study, the study design or type of data used, the key findings, as well as implications of the findings for future NCD research were extracted from each study. The data extraction was carried out by the first reviewer (AS) and cross-checked by a second reviewer (JK) to ensure consistency and transparency in the process. At all points where discrepancies arose, all reviewers were involved in resolving them before the extraction continued.

### 2.5 Data synthesis

A narrative synthesis approach was employed for the analysis of the included studies. Our analysis procedure followed the framework suggested by Popay et al. [34] for the conduct of narrative synthesis and systematic reviews. The main outcomes of interest were analyzed using tables, figures, and texts, highlighting the mechanisms between climate change or globalization and NCDs in Africa. The geographical locations of all the included studies were captured. This was followed by summarizing and reporting the key characteristics of each study in an evidence table. Subsequently, the major pathways between climate change or globalization and NCDs that emerged from the review were reported and discussed comprehensively.

### 2.6 Methodological quality assessment

The methodological quality of the included studies was assessed using the critical appraisal tools for analytical cross-sectional studies and qualitative research proposed by the Joanna Briggs Institute (JBI) [35, 36]. These tools were employed due to the cross-sectional design and qualitative nature of the reviewed studies. The quality assessment was considered appropriate for minimizing potential bias (see **S2 and S3 Tables**).

The first author (AS) carried out the quality assessment and the results were subsequently verified by all authors. In sync with the JIB’s checklist, the quality assessment for each study was based largely on the clarity of inclusion and exclusion criteria, adequate description of the participants and the setting of the study, statement of strategies adopted to deal with confounding factors and the use of appropriate statistical analysis method.

## 3. Results

The results are organized into two broad sections. The first section reports findings from the search outcome and the characteristics of the studies included in the systematic review. The second section focuses on the relationship between globalization and or climate change and NCDs.

### 3.1 Search outcome

**Fig 2:**
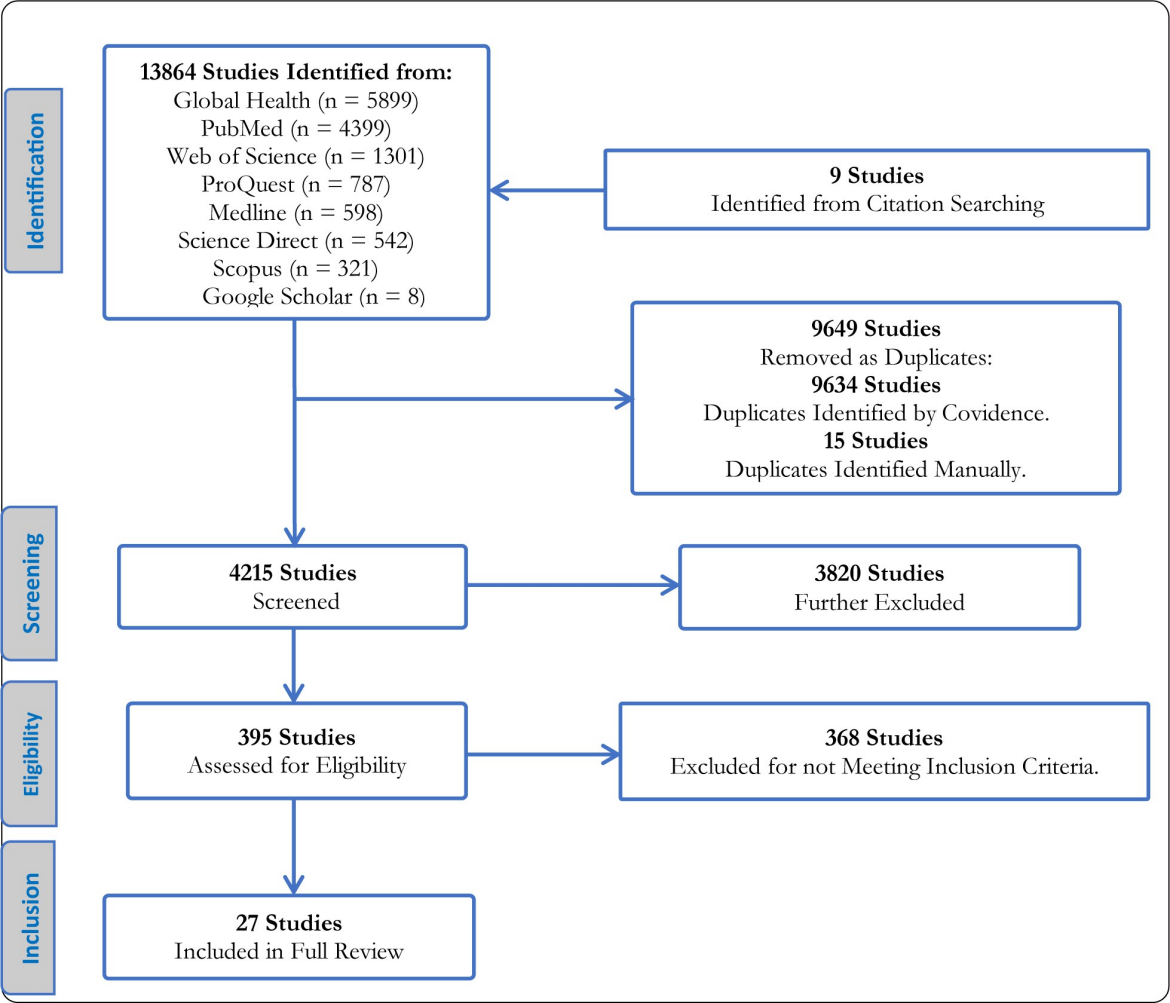
The search flowchart [32].

A total of 13864 studies were identified from the eight electronic databases, as shown in **Fig 2.** The following details were retained for each study: paper title, authors, journal name, volume and issue number, page numbers, and the abstract.

Of the 13864 studies identified, 9649 studies were removed as duplicates by a match of the titles. Another 3820 studies were excluded after the title and abstract screening for relevance, leaving 395 studies for full-text review. After the full-text review, 368 studies were further excluded for not meeting the inclusion criteria. Finally, 27 studies met the full eligibility criteria and were retained for further assessment.

### 3.2 Characteristics of the included studies

In-depth characteristics of the included studies are shown in **Table 1**. As earlier mentioned in **Section 2.4**, **Table 1** highlights the author(s), country of research, year of publication, study design, key findings, and implications of the findings of each study for future NCD research. The studies were published between 1996 and 2023. The studies covered 10 specific countries in 27 different locations: Cameroon [37], Ethiopia [38–40]); Kenya [19, 41–43], Namibia [20], Nigeria [27, 44], Senegal [45, 46], Sierra Leone [28], South Africa [47–50], Tanzania [51] and Uganda [52]. Four of the studies were generic, focusing on sub-Saharan Africa [53–56], while one study focused on multiple countries, specifically Cameroon, Kenya, Nigeria, and South Africa [15].

**Table 1:** Characteristics of included studies.

**A. CROSS-SECTIONAL (QUANTITATIVE STUDIES)**					
**Domain**	**Citation**	**Country/ Region**	**Focus of the Study**	**Key Findings**	**Implication for NCDs/Research Need**
Globalization	[47]	South Africa	Prevalence and contextual Correlates of NCDs in Migrants and Non-migrants Populations	More migrants (19.81%) than non-migrants (16.69%) reported prevalence of NCDs.	Need for increased education and awareness about NCDs, to change lifestyles about smoking and physical inactivity.
Globalization	[57]	Kenya	Patterns of unhealthful dietary, PA and sleep behaviors.	Prevalence of unhealthful behaviors varied significantly by age, gender, school income levels, city, and frequency of consumption of restaurant foods.	Need for enhanced childhood obesity prevention measures in Kenya and neighboring countries.
Globalization	[19]	Kenya	Effects of purchasing food in supermarkets on NCDs	Purchasing food in supermarkets had an increased probability of being obese, and a higher likelihood of suffering from pre-diabetes.	Effects of supermarkets on nutrition and health can mainly be ascribed to changes in the composition of people’s food choices.
Globalization	[42]	Kenya	Behavioral risk factors of common NCDs slum dwellers.	One out of five of people in the urban slum settings of Nairobi had co-occurrence of NCD risk factors.	Comprehensive & differentiated approaches needed for effective NCD prevention.
Globalization	[52]	Uganda	Alcohol use among adults and NCDs.	High level of alcohol use causes alcohol-use-related NCDs.	The need to control the intensive intake of alcohol to reduce the prevalence of NCDs.
Globalization	[20]	Namibia	Dietary patterns and NCDs.	High uptake levels of starch–sugar–oil diets are associated with diabetes.	Context specific interventions required to address dietary related NCDs.
Globalization	[38]	Ethiopia	Diet-related behaviors & NCDs.	Dietary behavior contributes significantly to NCD burdens. Intakes of diet low in fruits and vegetables and high in sodium are the leading dietary risks.	Multisectoral interventions are required to effectively mitigate the burden of NCDs.
Globalization	[39]	Ethiopia	NCDs and associated factors among working adults.	Overall, 95.8% of the participants had at least one of the five risk factors of NCDs.	The need for lifestyle modification and comprehensive NCDs prevention programs.
Globalization	[43]	Kenya	Dietary risk factors for NCDs.	Prevalence of high reported dietary salt intake and sugar associated with NCDs.	Further research is necessary to understand the knowledge: practice mismatch on unhealthy diets.
Globalization	[45]	Senegal	NCDs and Risk Factors	The risk of high cholesterol was 2.42 in the 35–44 age group and 2.86 in the 45–60 age group in comparison with the 25–34 age group. 32% were classified as having stage 2 chronic kidney.	Intervention for the prevention of NCDs and enhance health promotion is needed.
Globalization	[48]	South Africa	Food consumption expenditure & NCDs	Real food expenditure on certain foods exacerbates the burden of diet related NCDs.	Reducing the cost of access to nutritious foods for the prevention of NCDs may be useful.
Globalization	[46]	Senegal	Red meat consumption & NCDs	High consumption of red meat increases the risks for NCDs.	The need for educational intervention on the controlled use of meaty (proceed meat) foods.
Climate change	[49]	South Africa	Migration, Urban living and NCDS.	Migration negatively affects women’s health through higher blood pressure (BP).	The need for further interrogation of the ways in which migration and urban living are structured by gender in LMICs.
Globalization	[28]	Sierra Leone	Ebola and NCDs	Comprehensive reporting of NCDs was suboptimal and declined during the Ebola epidemic.	The need to put in adequate measure to ensure that epidemics such Ebola do not unduly overburden the severity of NCDs.
Globalization	[50]	South Africa	Risk factors for NCDs and association with the ’Healthy choices at work’ program.	The HCWP was associated with clinically significant reductions in behavioral, metabolic, and psychosocial risk factors for NCDs.	Intervention to promote the making of health-friendly decisions at workplaces may be useful for the prevention of NCDs.
Globalization	[15]	(Cameroon, Kenya, Nigeria, and South Africa)	Globalization processes, the food environment, and dietary health outcomes in SSA.	Higher profit margins on processed foods have promoted the creation of ‘obesogenic’ environments.	Further research may be needed to enhance the understanding of the impacts of globalization processes on dietary health outcomes in SSA.
Globalization	[51]	Tanzania	Neighborhood clustering of NCDs.	Among NCDs, hypertension exhibited the strongest clustering within neighborhoods followed by chronic kidney disease (CKD), obesity and glucose impairment.	The results may help inform the design of future community-based studies examining NCDs.
**B. QUALITATIVE STUDIES**					
**Domain**	**Citation**	**Country/ Region**	**Focus of the Study**	**Key Findings**	**Implication for NCDs/Research Need**
Climate change	[53]	Sub-Saharan Africa	Impact of climate change and urbanization in Africa	Climate change and urbanization are putting additional pressure on unplanned urban food systems across Sub-Saharan Africa.	Need for research to further understand the link between climate change, urbanization, and fast-food retailing.
Globalization	[54]	General	Global Environmental Change (GEC) and NCD Risks	Risk Factors for global GEC Influence NCD risk through a range of mechanisms.	Need for further research on the direct and in-direct impact of GEC on NCDs
Climate change	[55]	General	Global climate change impacts NCDs.	Chronic disease risks are likely to increase with climate change and related increase in malnutrition, and extreme weather events.	Further research needed to guide the monitoring of the health impacts of climate change on NCDs
Globalization	[44]	Nigeria	Burden of NCDs) & globalization.	The burden of NCDs is on the rise in Nigeria due to various forces of globalization	Efforts should be targeted at strengthening health systems to address NCD prevalence.
Globalization	[48]	South Africa	Food consumption expenditure & NCDs	Real food expenditure on certain foods exacerbates the burden of diet related NCDs.	Reducing the cost of access to nutritious foods for the prevention of NCDs may be useful.
Globalization	[59]	Africa	Transitioning food environments and diets of African migrants	Dietary practices of African migrants are not homogenous.	There is the need to recognize the heterogeneity of the dietary practices of African migrants.
Globalization	[27]	Nigeria	Prevalence & knowledge of NCDs.	Overall, 99.3% of respondents had at least one behavioral risk factor. Behavioral risk factors for NCDs were prevalent among undergraduates.	Need to enhance knowledge and understanding about NCDs.
Globalization	[37]	Cameroon	Urbanization, Risk Profiles for Non-NCDs.	Urbanization was associated with a less active lifestyle and a dietary pattern that was higher in fat and lower in fruit and vegetable intake.	The need to identify children with unfavorable risk profiles to modify their behavior towards minimizing the burden of NCDs.
Climate change	[60]	Sub-Saharan Africa	Climate change and NCDs	Burden of NCDs is associated with climate changed induced urbanization in SSA.	Urgent need for climate sensitive NCD indicators in SSA policies, adaptation plans.
Globalization	[56]	General	Environmental roots of NCDs and the epigenetic impacts of globalization.	Epigenetic modifications related to globalization is associated with patterns of NCDs.	Interventions are required to address the negative contribution of globalization on NCDs.

**Fig 3:**
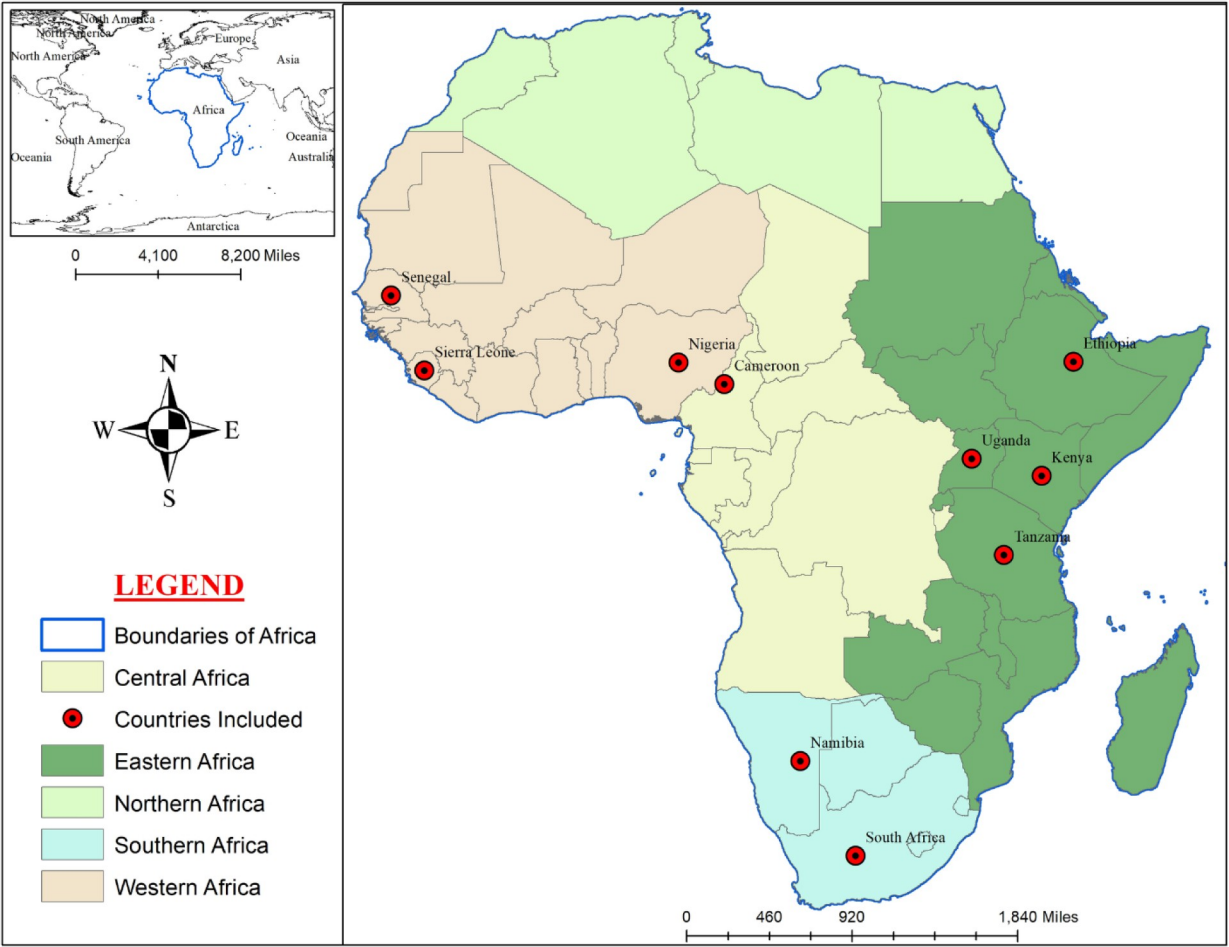
Regional distribution of included studies.

The regional—Central, Eastern, Northern, Sothern and Western—distribution of these countries is shown in **Fig 3**. The studies used both primary [19, 20, 28, 37, 39, 40, 42, 45, 46, 50, 51, 57] and secondary datasets [19, 38, 43, 44, 47–49, 52], drawn from cross-sectional surveys using structured and semi-structured questionnaire instruments (e.g., focus group discussions and in-depth interviews) [19, 20, 28, 37, 39, 40, 42, 45, 46, 51, 57] as well as national datasets such as the NCDs risk survey data of Uganda for 2014 [52]; the Urban Kenya demographic, anthropometric, and bio-medical data [19], the Kenyan STEP 2015 survey data [58] and the South African National Income Dynamics Study (NIDS) wave 5 of 2017 data [47]. Predominantly, the studies employed both qualitative [27, 37, 44, 48, 53–56, 59, 60] and quantitative [15, 19, 20, 28, 38–40, 42, 43, 45–47, 49–52, 57] approaches in the analyses of the datasets, focusing on the influence of climate change [49, 53, 55, 60] and globalization [15, 19, 20, 27, 28, 37–40, 42–48, 50–52, 54, 56, 57, 59] related factors on NCDs prevalence in Africa.

Together, the studies had a large pool of participants, ranging from 119 [37] (in Cameroon) to over 28055 [47] (in South Africa) participants, consisting of both males and females, aged between 10 and 70+ years. NCD risk factors varied across socio-demographic attributes, suggesting that people living in the highest wealth quintile are more likely to have higher prevalence of NCDs such as type II diabetes compared to other cohorts [58]. The significant risk factors for NCDs identified from the studies include physical inactivity [37, 50], tobacco smoking and harmful alcohol consumption [37, 50, 52]), overweight [57], obesity [51, 57] and raised blood pressures [51, 61].

The methodological quality assessment of the included studies is also shown in **S2 Table**. Overall, the assessment revealed a moderate risk of bias but did not provide strong evidence to doubt the results reported. Importantly, the objectives that underpinned the studies were clearly stated as well as descriptions of the study populations and how the samples were drawn. In each study, a reasonable proportion of the eligible population was recruited using scientifically rigorous methods of sample selection process guided by sound inclusion and exclusion criteria. Also, different statistical analysis methods were employed to identify and account for the influence of single or multiple potential confounders [45, 46, 51]. However, some of the studies [28, 42, 47, 50] did not clearly state how confounders were identified and how they were accounted for in their analysis, which may have moderately biased the findings of the studies. Likewise, the cross-sectional nature of the included studies did not allow sufficient time to assess the causal relationships between the risk factors for NCDs and the prevalence of NCDs among the samples. Essentially, most of the studies [19, 20, 28, 37, 39, 40, 42, 45, 46, 51, 57] relied on one-time retrospective self-report of the participants using a questionnaire, which made it difficult to discern the accuracy of the reports.

### 3.3 The climate change, globalization and NCDs nexus

The Intergovernmental Panel on Climate Change (IPCC) defines climate change as ‘a change in the state of the climate measured by changes in the mean or the variability of its properties, which may persist for a long period of time’ [62] [p.120]. Globalization, on the other hand, is arguably a term that is still subject to different views in terms of its meaning and influence on human life [19]. Nonetheless, globalization is generally considered as a phenomenon that involves the spatial restructuring of relatively free and smoother trans-border movement of goods and services [63], and as a socio-cultural process, involving the diffusion of cultural ideologies and sensitivities [19]. This understanding suggests the concept of globalization as synonymous with Westernization, considering that globalization has been at the forefront of the adoption of Western ways of living, especially within the context of changing dietary lifestyles and socio-cultural behaviors [20, 38].

**Fig 4:**
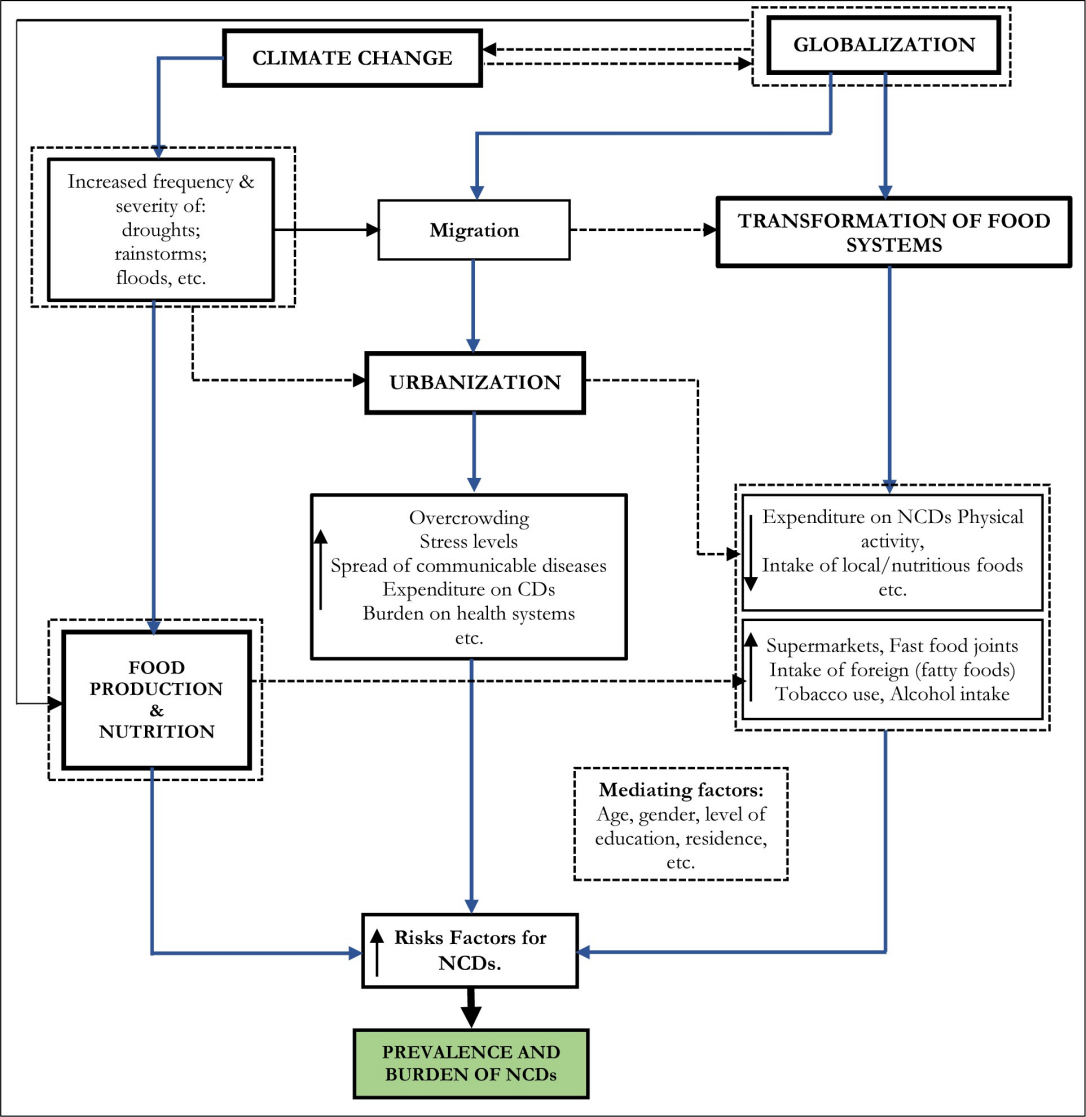
Relationship between climate change, globalization and NCDs.

Three major pathways, through which climate change and or globalization interact with NCDs, emerged from the review. As presented in **Fig 4**, these pathways are: (a) food production and nutrition (b) urbanization; and (c) transformation of food systems. An overview of these pathways is presented in **Table 2** while each pathway is discussed comprehensively in the next three sub-sections, respectively.

**Table 2:** An overview of climate change, globalization and NCD discourse.

Key Domain	Citation	Country(ies)	Type of NCD(s) linkage	Potential linkage with NCDs
Climate change, food production & nutrition	[53, 55, 60]	General (Sub-Sahara Africa), South Africa	Reduced food production and affordability of nutritious food; crop failure, food systems and nutrition insecurity.	Obesity/undernutrition (malnutrition), type-2 diabetes, cardiovascular disease, etc.
Globalization & urbanization	[37, 53, 56, 60]	General (Sub-Saharan Africa) Cameroon	Increased sedentary lifestyles, reduced active lifestyle/physical activity, increased intake of diet rich in fat, lower intake of fruit and vegetables, slums, stress, exposure to communicable diseases.	Overweight, obesity, raised blood pressures, type-2 diabetes, cardiovascular disease, etc.
Globalization & transformation of food systems	[15, 19, 20, 38, 44, 48, 53, 57, 59]	General (Sub-Saharan Africa), Cameroon, Kenya, Nigeria, and South Africa, Namibia, Ethiopia,	Increasing prevalence of unhealthy dietary behaviors (consumption of fast foods, alcohol, smoking, etc.), popularity of supermarkets (changes in the composition of people’s food choices, etc.).	Higher BMI, probability of being obese, higher likelihood of pre-diabetes, raised blood pressures, type-2 diabetes, cancer. etc.

#### 3.3.1 Climate change and food production and nutrition pathway

The issue of climate change and its impact on food production is increasingly recognized, not only in Africa, but also in different parts of the world [63]. In the context of this study, some [53, 55, 60] have discussed the influence of climate change on NCDs, through how climate change events affect food production, leading to food insecurity, increasing incidence of malnutrition and foreign food retailing. For instance, in 2007, the Intergovernmental Panel on Climate Change (IPCC) hinted that in about 50 years, agricultural productivity in Africa may decline by up to 12%, due to climate change effects [64]. In the context of Tanzania, Rowhani and colleagues also reported that by 2050, the projected increase of 2°C will reduce the average production of rice, sorghum and maize, which are major staple foods for most Africans, by 7.6%, 8.8% and 13%, respectively [65].

Although the occurrences of climate change events are not limited to Africa, Africa seems to be the embodiment of the vulnerability of climate change effects [66] as the continent continues to disproportionately receive the negative impacts of climate change-induced natural disasters [12]. For instance, between 1991 and 2021, Africa consistently recorded warming trends in temperatures, with an annual increase of about +0.3°C, higher than the average recorded (+0.2°C) for the period between 1961 and 1990 [67]. This led to about 34% reduction in agricultural productivity in Africa, which was markedly higher than any other region’s since 1961 [67]. Projections are suggesting that, if nothing is done, this trend may continue to hit most parts of Africa with further negative outcomes on food security, nutrition and health [68]. Consequently, it is argued that the current and the projected outlook of African climatic conditions may undermine the continent’s ability to achieve its targets of the Sustainable Development Goals (SDGs), target 3.4 (i.e., to reduce by one-third premature mortality from NCDs by 2030) [11].

The included studies have reported that droughts, for example, have been a leading cause of livelihood destruction in Africa for some time now. Drought is generally used to describe a prolonged period of dryness due to high temperatures and low rainfall [69]. Like most natural disasters, droughts are not unique to Africa, and several other countries outside Africa have also experienced droughts at one point in time or the other. However, Africa has historically witnessed several episodes of prolonged and extreme droughts across the continent, bringing about food insecurity, health disparity, morbidity, and mortality [70, 71].

For example, Qu and colleagues reported that, between 2010 and 2011, countries that are commonly referred to as the Horn of Africa (HOA)—Djibouti, Eritrea, Ethiopia, and Somalia—simultaneously experienced severe droughts, which led to severe degrees of food insecurity, famine, malnutrition and loss of several lives [72]. Similarly, approximately 3.7 and 2.6 million people in South Africa and Madagascar, respectively, were affected by droughts in 2019 [14, 73], which all led to severe food shortages, and consequently famine. The Western Cape of South Africa also experienced severe droughts during the period between 2015 and 2018 [74]. Such events caused dams to dry significantly, affecting the livelihoods of about 3.7 million people [13]. Ishumael and Godwell [75] also reported how communal farmers in Zimbabwe were exposed to unfamiliar drought conditions that increased their vulnerability to food insecurity and poverty. In projected terms, Habtemariam et al., [76] have argued that, by 2030, climate change-induced droughts may negatively affect small-scale farming activities more progressively than presently being experienced in Ethiopia. Increasing frequency and severity of drought, as a climate change outcome, has many health implications [70, 71]. As shown in **Fig 4,** drought can negatively affect crop production [75]. In many parts of Africa, low crops, and vegetable production due to droughts have caused food shortages and led food prices to rise, potentially increasing reliance on processed foods [65, 77, 78]. Similarly, during droughts, livestock raised for food can become malnourished and can even die in severe cases [79], potentially increasing the risk for NCDs due to limited access to essential food nutrients.

Another key issue of climate change in Africa is rising temperatures in urban areas, which have presented unusual records of heat waves in the continent. Temperatures in the African continent have been warming at a faster pace than the world’s mean surface temperature [80]. For example, the annual average temperature for the year 2020 was 1.19°C above average, making 2020 the fourth warmest year in the African continent since the last century (precisely since 1910) [81]. It is forecasted that, towards the end of the 21^st^ century, temperatures in extensive parts of Africa may exceed 2°C of the expected average warming [82]. The effects include reduced agricultural productivity, lower dietary diversity, and higher food prices [83], putting people at higher risk of many NCDs [84].

Severe heat waves adversely affect agriculture activities, crop production and nutrition intake [82, 85] and public infrastructure development and management, which are all essential for promoting the prevention of NCDs [86–88]. Africa is relatively more vulnerable to heatwaves mainly because of its limited adaptation expertise exacerbated by ineffective institutional structures to effectively manage the outcomes of heatwaves [89]. Heat stress causes accelerated blood circulation and heart rate, and changes in blood pressure levels., which can lead to insufficient cardiac output to fulfill the thermoregulatory needs of the human body [90]. Excessive temperatures can worsen the health status of individuals suffering from diabetes because their skin’s blood flow and sweating responses are lower compared to that of healthy individuals during heat exposure; also, excessive heat can be detrimental to an individual’s glycemic control, leading to glycemic shocks [91].

#### 3.3.2 Globalization and urbanization pathway

The pathway between globalization and urbanization on NCDs was captured through the indirect influence of migration (intra- and inter-national) on human health [92, 93]. Migration is associated with both benefits and harms. Migration may supply low-cost labor for host countries or cities and remittances from emigrant workers can be essential sources of income for many people [92]. Some have also argued that urban living through migration may improve the health conditions of people living with NCDs through enhanced access to better health services including access to essential and beneficial health information [94]. On the contrary, migration is conceived to be an underlying determinant of NCDs as it is commonly associated with increased exposure to behaviors that contribute to the tendency of becoming more prone to NCDs [95].

The studies argue that in the context of Africa, immigration has induced the formation of slum, leading to overcrowding, stress, and exposure to communicable and non-communicable diseases and urban food environments, as immigrants are often forced to sleep in crowded rooms with limited access to quality water and hygiene services in their neighborhoods [27, 42]. Thus, urban living significantly influences the exposure of migrants to the risk factors of NCDs such as poor dietary changes [20, 38, 59], increased smoking and alcohol consumption and stressful living conditions [43]. Cities also tend to be devoid of green and open space for outdoor physical activity, coupled with higher prevalence of crime, violence, and threat on human life [50].

Pheiffer [49], for instance, examined the influence of internal migration and urban living on the risk factor for NCDs in the context of South Africa. The author found that internal migration and urbanization continue to be pervasive demographic and socioeconomic phenomena that shape the daily living circumstances of South Africans. They also found that while urban residence appeared to convey a health advantage when men reside in urban areas compared with rural places, there was no evidence of an urban health advantage among women. Instead, migration negatively affected women’s health through higher blood pressure (BP). Earlier, Proctor et al., [37] examined the risk profiles for NCDs in rural and urban school children in the Republic of Cameroon. They found that physical activity among rural children was more than twice that of urban children. Also, they found that rural children consumed fewer foods containing fat and more fruits and vegetables relative to their counterparts in urban areas. Their overall conclusion was that urbanization was associated with a less active lifestyle and dietary pattern that was higher in fat and lower in fruit and vegetable intake. Ajaero et al., [47] posited that changing lifestyles were also associated with migration and urbanization and led to increased reported levels of stress and reduced physical activity levels.

#### 3.3.3 Globalization and transformation of food systems

The second pathway through which globalization impacts NCDs is reflected in the way it contributes to changing food systems in Africa. Food transformation is the shift in dietary patterns that influences human health [96]. While several factors are noted to be underpinning food shifts, climate change and globalization are at the forefront [18, 55]. One of the central shifts that has occurred in the global food system is the increasing disappearance of fresh food markets in most parts of Africa and traditional fruit trees [41]. Most African countries, for instance, are fast becoming flooded with multinational and regional supermarkets. Thus, purchasing food from supermarkets has also been spreading across capital cities to rural areas in Africa [19, 20]. Some scholars, e.g., [21], are of the view that development in the fast-food industry in the developed world has led to the shifting pattern of dietary behavior in the developing world, especially in Africa. For instance, Coca-Cola products are now sold in over 200 countries around the world, while the records of fast-food restaurants such as KFC, McDonald’s, Pizza Hut, and Kentucky’s Fried Chicken restaurants, are also increasing in most parts of Africa [15, 97].

Various studies suggest that, in relation to dietary behavior, as the transition from reliance on the ability to produce food to reliance on the ability to purchase food increases, people eat what is available and cheap. To some extent, this has led the global fast-food industry to bring to African markets energy-dense products that are nutritionally poor but attractively priced and marketed [20, 37, 38]. In the context of Kenya, Demmler et al., [19] investigated the impact of supermarket food purchases on nutrition related NCDs, focusing on the direct effect on people’s BMI, as well as on health indicators such as fasting blood glucose (FBG), blood pressure (BP), and the metabolic syndrome. The authors found that supermarkets and their food sales strategies seem to have directly affected people’s health, suggesting that, in addition to increasing overweight and obesity, supermarkets contributed to nutrition related NCDs. Modernization in the food retail sector is typically associated with changes in the types of foods offered, prices, packaging sizes, and shopping atmosphere. Remarkably, more prevalent than it was in the past four decades or so, food consumers in urban Africa now buy their foods in supermarkets but not from traditional retail stores [98, 99].

The above findings have been corroborated in other studies, providing additional evidence that traditional diets are being abandoned in favor of a more Western diet in both urban and rural Africa [20, 45]. Kazembe et al., [20], for instance, investigated the relationship between dietary patterns and NCDs in Windhoek, Namibia based on data from a cross-sectional random sample of 863 households. The authors identified three major dietary patterns: starch–sugar–oil, fruits–vegetables, and meat–fish, which explained more than 43% of the variation in food consumption. They observed that high uptake levels of starch–sugar–oil diets were associated with diabetes and increased heart problems. The prevalence of diabetes and hypertension differed significantly with household dietary diversity. Both were more common in households with higher dietary diversity, which is likely a function of higher levels of consumption of sugars and processed foods [100].

Relatedly, the studies also discussed the globalization of the beverage/tobacco industry in Africa and its associated impacts on NCD risk factors. In the context of Nigeria, Maiyaki and Garbati, [44] argued that globalization has created an enabling environment for expansion of both alcohol and tobacco markets, potentially influencing the creation of a growing culture that associates consumption of such products with high social status. Motuma et al., [39] in the context of Ethiopia, and Kabwama [52] in the context of Uganda also acknowledged the phenomenon. These authors argued that increasingly exposing vulnerable populations, especially the youth, to alcohol and tobacco consumption, has the potential of expanding the base of NCD epidemic in Africa [44].

## 4. Discussions

This review aimed to document the pathways through which climate change and or globalization influence non-communicable diseases (NCDs) in Africa. In general, we found that the impacts of climate change and or globalization on NCDs are mainly indirect, acting through different pathways that influence the prevalence and the burden of NCDs in Africa. Specifically, three pathways emerged from the review, suggesting that while climate change indirectly influenced NCDs through reduction in food production and the intake of nutritious foods in the African continent, globalization influenced NCDs through urbanization and the transformation of food systems.

The studies have demonstrated that in the last three decades, there has been an accelerated change in the diets and lifestyles of populations around the African continent. This evolution is mainly linked to the pressures of urbanization and the growing globalization of traditional food systems [46]. The findings from the studies showed that the patterns of food consumption in Africa have tremendously changed from the consumption of local foods from traditional food markets to the consumption of foreign foods from supermarkets, which have relatively higher energy density and are generally rich in saturated fat, salt, and sugar, with less unrefined carbohydrates [19].

The consumption of meat, for instance, was identified in most of the studies as an aspect of the dietary pattern that has changed over the years, being a primary risk factor for NCDs [19, 20, 46, 57]. Previous studies have argued that, although meat makes up only about 10% of global food mass and energy, it delivers most of the vitamins needed by humans [101, 102]. However, what has been observed in this study is an increased consumption of red/processed meat. Wolk’s [103] study, for instance, revealed that the consumption of red meat (100 g/day) and processed meat (50 g/day) was associated with an increased risk of diabetes, coronary heart disease and stroke. This finding collaborates with findings from some recent studies such as the study of Giromini and Givens, [102]. Giromini and Givens have advanced evidence to suggest that the consumption of red or processed meat, as opposed to white meat, is associated with increased risks of cardiovascular diseases (CVDs) and cancer. Similarly, in their study of the determinants of intention to Consume Dibiterie Meat towards the Risks of NCDs in the Dakar Region of Senegal, Orou Seko et al., [46] also observed a transition from consuming traditional foods to foreign foods, which were previously not common in Senegal. The authors attributed such transition to an observed increase in the pattern of out-of-home consumption, particularly in working-class neighborhoods, and in the development of collective catering workshops called “dibiterie”. Dibiteries offer braised sheep meat that is particularly popular and anchored in the eating habits of populations more exposed to the risk of NCDs [46].

Changes in job and labor culture over the last four decades have also been marked as aspects of the sociocultural fabric of Africans that have had some influence on NCDs in Africa. Hence, the reported lifestyle and associated risk factors for NCDs in most of the studies were generally attributed to such transformations. For instance, in the context of Ethiopia, Yosef [40] assessed the prevalence and associated factors of chronic NCDs involving 422 cross-country truck drivers. The author found an overall prevalence of NCDs among the participants to be 28.5%. This finding is in line with previous studies, e.g., [104, 105], which have also argued that truck driving is a job that exposes drivers to more risk factors for NCDs than other jobs. While driving is not an entirely new development in Africa, driving trucks for long hours across cities and countries is a relatively recent development due to the influence of the globalization of goods and services. Driving for long hours, as a professional occupation, is a typical sedentary behavior that can be considered a recent socio-cultural practice in most parts of Africa. Indirectly, the workplace environment shapes employees’ health and safety, influences their health behaviors through physical and psycho-social mechanisms and can influence the risk of developing NCDs [50]. Occupational psychosocial stress factors such as high workload (e.g., driving for long hours), psychological demands and lack of social support, are all associated with employees’ health and psychological well-being [106]. Therefore, it is not entirely surprising that the transformation of the sociocultural environment, including dietary patterns, has been discussed in the included studies as contributing to the prevalence of NCDs in Africa.

Another significant influence of globalization on NCDs reported in some of the studies [27] is the indirect influence of globalization on the spread of emerging and re-emerging infectious diseases including COVID-19 in the African continent. It is believed that the impact of COVID-19 would not have been such severe, but for the seamless possibility for people to travel and for goods and services to be fast transported across borders. Apparently, the battle against COVID-19 placed pressure on scarce resources, leading to an increased healthcare burden for patients with NCDs as resources were repurposed for the acute problem of COVID-19 pandemic control [27]. Owopetu et al., [27] assessed the implications of COVID-19 and the continuity of care for NCDs in sub-Saharan Africa. The authors found that in the context of the COVID-19 pandemic, many people living with NCDs found accessing care more difficult. They added that the majority of the available resources on the African subcontinent were diverted to focus on the COVID-19 pandemic, thereby causing interruptions in care, complication in management, and drug pick-up, to the point of almost neglecting care for NCDs [27]. Also, in many other countries in Africa, patients with single or multiple NCDs found themselves with no or unpredictable/disrupted clinic follow-up visits for weeks or months and drug pick-ups at some locations became inaccessible [107]. These constraints, combined with an already resource-limited health system, led to increased morbidity and mortality for patients with NCDs in the African region [27, 107].

In the context of Sierra Leone, Samba et al., [28] found another interesting way through which globalization impacted the spread of the Ebola virus and how that in turn affected the management of NCDs in the country. The aim was to describe reporting systems, monthly attendances, and facility-based patterns of six NCDs, including hypertension, cardiovascular diseases, diabetes, and peptic ulcer, in the pre-Ebola and during the Ebola virus disease outbreak periods. Overall, the authors observed a marked decline in the reporting of NCDs during the Ebola period, with a monthly mean of 342 cases pre-Ebola and 164 during the Ebola outbreak. This observation is similar to Edelstein and colleagues’ [108] comments regarding how the Ebola virus disease (EVD) influenced the risk for some communicable and non-communicable diseases in Ebola-affected countries in Africa due to a decline in hospital attendance, and or case reporting. Similarly, Samba and colleagues are of the view that, the downward reporting trends observed for most NCDs during the Ebola disease outbreak could be attributed to a decrease in clinic attendance, as well as the closure and repurposing of some health facilities for the Ebola response. It is also likely that the redeployment of skilled human resources and the tragic deaths of healthcare workers due to Ebola worsened some NCD conditions.

## 5. Limitations and conclusion

This systematic review has several limitations that should be considered while acknowledging the findings. First, we excluded non-English studies as the authors do not speak or understand these other languages and do not want to misinterpret or misrepresent these studies. Thus, we may have excluded some relevant studies published in other languages. Further, despite using a comprehensive search strategy to complete our electronic search in eight electronic databases, it is still likely we may have missed additional relevant articles that may have contributed further insights to our analysis. It is also important to state that a vast majority of the included studies used cross-sectional survey data. While the studies have demonstrated sound theoretical bases for their study designs, we acknowledge that cross-sectional data do not provide definitive assertions of causality. Thus, the arguments from the included studies may not be unbiased of the true picture of NCDs and the interrelationships of NCDs with climate change and or globalization in the context of Africa. Despite these limitations, we believe that we had a strong scientific base supporting the inclusion of the studies reviewed in this paper.

In summary, the findings of this systematic review suggest that climate change and globalization are undeniably at the forefront of the prevalence of NCDs, putting additional financial and social anxiety on the containment of NCDs in Africa. Climate change negatively impacts food production and nutrition through increasing frequency and severity of droughts, heatwaves, rainstorms, floods, and other severe weather events that are not conducive to food production for the African population. Globalization, on the other hand, induces transformation in food and sociocultural systems and influences urbanization, leading to overcrowding, stress levels, and the spread of communicable diseases, especially in urban Africa. The connection between climate change and globalization is demonstrated in the way their direct and indirect effects contribute to reducing expenditure on NCDs (due to a higher focus on communicable diseases such as HIV/AIDs, Ebola, COVID-19, etc.), decreasing physical activity level and decreasing the intake of local/nutritious foods. The review further suggests that the issues influencing NCDs in Africa, through the forces of climate change and globalization, are complex, and depend on several interrelated economic, social, cultural, and environmental factors.

Overall, our review showed that there are more studies or literature on globalization and NCDs linkage relative to the influence of climate change on NCDs. This indicates that in the context of Africa, much research attention has not been given to the impact of climate change on NCDs. Thus, while the health sector would have a major role to play in enabling further research and monitoring the impacts of climate change on NCDs, researchers would also need to examine the specific pathways between global climate change and NCDs toward appropriate interventions for the control of NCDs in Africa. It may also be useful for future studies to explore the management practices that people afflicted with NCDs are employing to cope with their conditions, as well as how they are adapting to the changing food systems and nutrition under the influence of climate change and globalization [109]. In doing so, it may be essential to recognize the pervasiveness of the economic, social, and political forces underpinning human behavior about food choices and dietary behaviors for context-appropriate health-related interventions [109].

## Supporting information

S1 Table Key search strategy. (DOCX)

S2 Table PRISMA checklist of systematic review. (DOCX)

S3 Table PRISMA 2020 checklist. (DOCX)

## References

[1] Benziger CP, Roth GA, Moran AE. The global burden of disease study and the preventable burden of NCD. Global heart. 2016;11(4):393–7. doi: 10.1016/j.gheart.2016.10.024 27938824

[2] Intergovernmental Panel on Climate Change [IPCC]. Climate Change 2022: Impacts, Adaptation, and Vulnerability. Contribution of Working Group II to the Sixth Assessment Report of the Intergovernmental Panel on Climate Change. 2022. [Pörtner H.-O., Roberts D.C., Tignor M., Poloczanska E.S., Mintenbeck K., Alegría A., Craig M., Langsdorf S., Löschke S., Möller V., Okem A., Rama B. (eds.)]. Cambridge University Press. Cambridge University Press, Cambridge, UK and New York, NY, USA, 3056 pp., doi: 10.1017/9781009325844

[3] Swinburn BA, Kraak VI, Allender S, Atkins VJ, Baker PI, Bogard JR, et al. The global syndemic of obesity, undernutrition, and climate change: the Lancet Commission report. The lancet. 2019;393(10173):791–846. doi: 10.1016/S0140-6736(18)32822-8 30700377

[4] Terzic A, Waldman S. Chronic diseases: the emerging pandemic. Clinical and translational science. 2011;4(3):225. doi: 10.1111/j.1752-8062.2011.00295.x 21707955 PMC5439863

[5] World Health Organization [WHO]. Noncommunicable diseases. 2022. Retrieved March 31, 2023, from https://www.who.int/news-room/fact-sheets/detail/noncommunicable-diseases.

[6] UN SDGS 2020: The United Nations Sustainable Development Goals. Retrieved from: <https://sdgs.un.org/goals/goal3 on 20/02/2023>.

[7] Allen LN, Pullar J, Wickramasinghe KK, Williams J, Roberts N, Mikkelsen B, et al. Evaluation of research on interventions aligned to WHO ‘Best Buys’ for NCDs in low-income and lower-middle-income countries: a systematic review from 1990 to 2015. BMJ global health. 2018;3(1): e000535. doi: 10.1136/bmjgh-2017-000535 29527342 PMC5841523

[8] Gonçalves RL, Pagano AS, Reis ZSN, Brackstone K, Lopes TCP, Cordeiro SA, et al. Usability of Telehealth Systems for Noncommunicable Diseases in Primary Care From the COVID-19 Pandemic Onward: Systematic Review. Journal of medical Internet research. 2023;25: e44209. doi: 10.2196/44209 36787223 PMC10022651

[9] World Health Organization [WHO]. Noncommunicable diseases (NCDs): Key facts. 2018. Retrieved from https://www.who.int/news-room/fact-sheets/detail/noncommunicable-diseases.

[10] Ezzati M, Pearson-Stuttard J, Bennett JE, Mathers CD. Acting on non-communicable diseases in low-and middle-income tropical countries. Nature. 2018;559(7715):507–16. doi: 10.1038/s41586-018-0306-9 30046068

[11] Nugent R, Bertram MY, Jan S, Niessen LW, Sassi F, Jamison DT, et al. Investing in non-communicable disease prevention and management to advance the Sustainable Development Goals. The Lancet. 2018;391(10134):2029–35. doi: 10.1016/S0140-6736(18)30667-6 29627167

[12] Abass K, Dumedah G, Frempong F, Muntaka AS, Appiah DO, Garsonu EK, et al. Rising incidence and risks of floods in urban Ghana: Is climate change to blame? Cities. 2022; 121:103495.

[13] Abiodun BJ, Makhanya N, Petja B, Abatan AA, Oguntunde PG. Future projection of droughts over major river basins in Southern Africa at specific global warming levels. Theoretical and Applied Climatology. 2019; 137:1785–99.

[14] Haile GG, Tang Q, Sun S, Huang Z, Zhang X, Liu X. Droughts in East Africa: Causes, impacts and resilience. Earth-science reviews. 2019; 193:146–61.

[15] Schram A, Labonté R, Sanders D. Urbanization and international trade and investment policies as determinants of noncommunicable diseases in Sub-Saharan Africa. Progress in cardiovascular diseases. 2013;56(3):281–301. doi: 10.1016/j.pcad.2013.09.016 24267436 PMC7111622

[16] Burrows K, Kinney PL. Exploring the climate change, migration and conflict nexus. International journal of environmental research and public health. 2016;13(4):443. doi: 10.3390/ijerph13040443 27110806 PMC4847105

[17] Popkin BM, Adair LS, Ng SW. Global nutrition transition and the pandemic of obesity in developing countries. Nutrition reviews. 2012;70(1):3–21. doi: 10.1111/j.1753-4887.2011.00456.x 22221213 PMC3257829

[18] Savage A, McIver L, Schubert L. the nexus of climate change, food and nutrition security and diet-related non-communicable diseases in Pacific Island Countries and Territories. Climate and Development. 2020;12(2):120–33.

[19] Demmler KM, Klasen S, Nzuma JM, Qaim M. Supermarket purchase contributes to nutrition-related non-communicable diseases in urban Kenya. PloS one. 2017;12(9): e0185148. doi: 10.1371/journal.pone.0185148 28934333 PMC5608323

[20] Kazembe LN, Nickanor N, Crush J. Food insecurity, dietary patterns, and non-communicable diseases (NCDs) in Windhoek, Namibia. Journal of Hunger & Environmental Nutrition. 2022;17(3):425–44.

[21] Lobstein T, Baur L, Uauy R. Obesity in children and young people: a crisis in public health. Obesity reviews. 2004; 5:4–85. doi: 10.1111/j.1467-789X.2004.00133.x 15096099

[22] Khraishah H, Alahmad B, Ostergard RL Jr, AlAshqar A, Albaghdadi M, Vellanki N, et al. Climate change and cardiovascular disease: implications for global health. Nature Reviews Cardiology. 2022:1–15. 10.1038/s41569-022-00720-x 35672485

[23] Jayasekara K, Kulasooriya P, Wijayasiri K, Rajapakse E, Dulshika D, Bandara P, et al. Relevance of heat stress and dehydration to chronic kidney disease (CKDu) in Sri Lanka. Preventive medicine reports. 2019;15:100928. doi: 10.1016/j.pmedr.2019.100928 31304082 PMC6603435

[24] Eze IC, Hemkens LG, Bucher HC, Hoffmann B, Schindler C, Künzli N, et al. Association between ambient air pollution and diabetes mellitus in Europe and North America: systematic review and meta-analysis. Environmental health perspectives. 2015;123(5):381–9. doi: 10.1289/ehp.1307823 25625876 PMC4421762

[25] Allen-Scott LK, Hatfield JM, McIntyre L, Rundall P. International travel and the globalization of health care: Implications for social work. Social Work in Health Care. 2014;53(9):854–69.

[26] McNairy ML, Tymejczyk O, Rivera V, Seo G, Dorélien A, Peck M, et al. High burden of non-communicable diseases among a young slum population in Haiti. Journal of Urban Health. 2019; 96:797–812. 31218502 10.1007/s11524-019-00368-y PMC6904710

[27] Owopetu O, Fasehun L-K, Abakporo U. COVID-19: implications for NCDs and the continuity of care in Sub-Saharan Africa. Global Health Promotion. 2021;28(2):83–6. doi: 10.1177/1757975921992693 33579179

[28] Samba T, Bhat P, Owiti P, Samuels L, Kanneh P, Paul R, et al. Non-communicable diseases in the Western Area District, Sierra Leone, before and during the Ebola outbreak. Public health action. 2017;7(1): S16–S21. 28744434 10.5588/pha.16.0086 PMC5515558

[29] Thomson MC, Muñoz ÁG, Cousin R, Shumake-Guillemot J. Climate drivers of vector-borne diseases in Africa and their relevance to control programmes. Infectious diseases of poverty. 2018;7(04):15–36. doi: 10.1186/s40249-018-0460-1 30092816 PMC6085673

[30] Schleussner C-F, Donges JF, Donner RV, Schellnhuber HJ. Armed-conflict risks enhanced by climate-related disasters in ethnically fractionalized countries. Proceedings of the National Academy of Sciences. 2016;113(33):9216–21. doi: 10.1073/pnas.1601611113 27457927 PMC4995947

[31] Ismael A, El-Gilany A. Pattern of skin diseases among Central African refugees in Chad. TAF Preventive Medicine Bulletin. 2015;14(4):324–8.

[32] Page MJ, McKenzie JE, Bossuyt PM, Boutron I, Hoffmann TC, Mulrow CD, et al. The PRISMA 2020 statement: an updated guideline for reporting systematic reviews. International journal of surgery. 2021; 88:105906. doi: 10.1016/j.ijsu.2021.105906 33789826

[33] Baatiema L, Chan CK, Sav A, Somerset S. Interventions for acute stroke management in Africa: a systematic review of the evidence. Systematic Reviews. 2017; 6:1–12. 29065915 10.1186/s13643-017-0594-4 PMC5655819

[34] Popay J, Roberts H, Sowden A, Petticrew M, Arai L, Rodgers M, et al. Guidance on the conduct of narrative synthesis in systematic reviews. A product from the ESRC methods programme Version. 2006;1(1): b92.

[35] Lockwood C, Munn Z, Porritt K. Qualitative research synthesis: methodological guidance for systematic reviewers utilizing meta-aggregation. JBI Evidence Implementation. 2015;13(3):179–87. 10.1097/XEB.0000000000000062 26262565

[36] Moola S, Munn Z, Tufanaru C, Aromataris E, Sears K, Sfetcu R, et al., (2020). Chapter 7: Systematic reviews of etiology and risk. In: Aromataris E, Munn Z (Editors). JBI Manual for Evidence Synthesis. JBI. 2020. Available from https://synthesismanual.jbi.global (Accessed on: May 20, 2023).

[37] Proctor MH, Moore LL, Singer MR, Hood MY, Nguyen U-SD, Ellison RC. Risk profiles for non-communicable diseases in rural and urban schoolchildren in the Republic of Cameroon. Ethnicity & disease. 1996;6(3/4):235–43. 9086313

[38] Melaku YA, Temesgen AM, Deribew A, Tessema GA, Deribe K, Sahle BW, et al. The impact of dietary risk factors on the burden of non-communicable diseases in Ethiopia: findings from the Global Burden of Disease study 2013. International Journal of Behavioral Nutrition and Physical Activity. 2016; 13:1–13. 27978839 10.1186/s12966-016-0447-x PMC5159959

[39] Motuma A, Demissie Regassa L, Gobena T, Teji Roba K, Berhane Y, Worku A. Almost all working adults have at least one risk factor for non-communicable diseases: Survey of working adults in Eastern Ethiopia. PloS one. 2022;17(2): e0264698. doi: 10.1371/journal.pone.0264698 35226698 PMC8884490

[40] Yosef T. Prevalence and associated factors of chronic non-communicable diseases among cross-country truck drivers in Ethiopia. BMC public health. 2020;20(1):1–7. 33069207 10.1186/s12889-020-09646-w PMC7568414

[41] Aworh OC. Food safety issues in fresh produce supply chain with particular reference to sub-Saharan Africa. Food Control. 2021; 123:107737.

[42] Haregu TN, Oti S, Egondi T, Kyobutungi C. Co-occurrence of behavioral risk factors of common non-communicable diseases among urban slum dwellers in Nairobi, Kenya. Global health action. 2015;8(1):28697. doi: 10.3402/gha.v8.28697 26385542 PMC4575413

[43] Mwenda V, Mwangi M, Nyanjau L, Gichu M, Kyobutungi C, Kibachio J. Dietary risk factors for non-communicable diseases in Kenya: findings of the STEPS survey, 2015. BMC Public Health. 2018;18(3):1–8. doi: 10.1186/s12889-018-6060-y 30400904 PMC6219002

[44] Maiyaki MB, Garbati MA. The burden of non-communicable diseases in Nigeria; in the context of globalization. Annals of African medicine. 2014;13(1):1–10. doi: 10.4103/1596-3519.126933 24521570

[45] Ndiaye AA, Tall AB, Gueye B, Fall IS, Seck SM, Mbodj AB, et al. A cross-sectional survey on non-communicable diseases and risk factors in the Senegalese Army. Health. 2016;8(14):1529–41.

[46] Orou Seko M, Laré N, Ossebi W, Fokou G, Dao D, Bonfoh B. Determinants of Intention to Consume Dibiterie Meat towards the Risks of Non-Communicable Diseases in the Dakar Region, Senegal. Sustainability. 2022;14(17):11000.

[47] Ajaero CK, Wet-Billings ND, Atama C, Agwu P, Eze EJ. The prevalence and contextual correlates of non-communicable diseases among inter-provincial migrants and non-migrants in South Africa. BMC Public Health. 2021;21(1):1–13. 34044795 10.1186/s12889-021-11044-9 PMC8161948

[48] Omotoso KO. Inequalities in Household Food Expenditures in South Africa: Implications for the Burden of Non-Communicable Diseases (NCDs) and Health Inequality. African Journal of Development Studies. 2022;12(4):225.

[49] Pheiffer CF. Internal migration, urban living, and non-communicable disease risk in South Africa. Social Science & Medicine. 2021; 274:113785. 10.1016/j.socscimed.2021.113785 PMC8041232 33684701

[50] Schouw D, Mash R, Kolbe-Alexander T. Changes in risk factors for non-communicable diseases associated with the ‘Healthy choices at work’programme, South Africa. Global Health Action. 2020;13(1):1827363. doi: 10.1080/16549716.2020.1827363 33076762 PMC7594846

[51] Stanifer JW, Egger JR, Turner EL, Thielman N, Patel UD. Neighborhood clustering of non-communicable diseases: results from a community-based study in Northern Tanzania. BMC public health. 2016;16(1):1–10. doi: 10.1186/s12889-016-2912-5 26944390 PMC4779220

[52] Kabwama SN, Ndyanabangi S, Mutungi G, Wesonga R, Bahendeka SK, Guwatudde D. Alcohol use among adults in Uganda: findings from the countrywide non-communicable diseases risk factor cross-sectional survey. Global health action. 2016;9(1):31302. doi: 10.3402/gha.v9.31302 27491961 PMC4974493

[53] Blekking J, Giroux S, Waldman K, Battersby J, Tuholske C, Robeson SM, et al. The impacts of climate change and urbanization on food retailers in urban sub-Saharan Africa. Current Opinion in Environmental Sustainability. 2022; 55:101169.

[54] Frumkin H, Haines A. Global environmental change and noncommunicable disease risks. Annual review of public health. 2019; 40:261–82. doi: 10.1146/annurev-publhealth-040218-043706 30633714

[55] Kjellstrom T, Butler AJ, Lucas RM, Bonita R. Public health impact of global heating due to climate change: potential effects on chronic non-communicable diseases. International journal of public health. 2010; 55:97–103. 19902143 10.1007/s00038-009-0090-2

[56] Vineis P, Stringhini S, Porta M. The environmental roots of non-communicable diseases (NCDs) and the epigenetic impacts of globalization. Environmental research. 2014; 133:424–30. doi: 10.1016/j.envres.2014.02.002 24593864

[57] Gewa CA, Onyango AC, Opiyo RO, Gittelsohn J, Cheskin LJ. Association between Primary School Children’s Unhealthful Behaviors and Overweight/Obesity: A Cross-Sectional Analysis in Urban Kenya. 2022.

[58] Mwangi KJ, Mwenda V, Gathecha G, Beran D, Guessous I, Ombiro O, et al. Socio-economic and demographic determinants of non-communicable diseases in Kenya: a secondary analysis of the Kenya stepwise survey. The Pan African Medical Journal. 2020;37. 10.11604/pamj.2020.37.351.21167 PMC7992900 33796165

[59] Osei-Kwasi H, Boateng D, Asamane EA, Akparibo R, Holdsworth M. Transitioning food environments and diets of African migrants: implications for non-communicable diseases. Proceedings of the Nutrition Society. 2023;82(1):69–79. doi: 10.1017/S0029665122002828 36453152

[60] Rother H-A. Controlling and preventing climate-sensitive noncommunicable diseases in urban sub-Saharan Africa. Science of the Total Environment. 2020; 722:137772. doi: 10.1016/j.scitotenv.2020.137772 32199361

[61] Nelson F, Nyarko K, Binka F. Prevalence of risk factors for non-communicable diseases for new patients reporting to Korle-Bu teaching hospital. Ghana medical journal. 2015;49(1):12–8. doi: 10.4314/gmj.v49i1.3 26339079 PMC4549813

[62] Intergovernmental Panel on Climate Change (IPCC). Climate Change 2014 Synthesis Report. IPCC: Geneva, Switzerland. 2014. Accessed from: https://www.ipcc.ch/site/assets/uploads/2018/02/SYR_AR5_FINAL_full.pdf (11/05/2023).

[63] Globalization Jenkins R., production, employment and poverty: debates and evidence. Journal of International Development. 2004;16(1):1–12.

[64] Intergovernmental Panel on Climate Change [IPCC]. Intergovernmental panel on climate change fourth assessment report, Cambridge University Press, Cambridge, UK. 2007.

[65] Rowhani P, Lobell DB, Linderman M, Ramankutty N. Climate variability and crop production in Tanzania. Agricultural and forest meteorology. 2011;151(4):449–60.

[66] Bwalya M. Comprehensive Africa Agriculture Development Programme (CAADP) to reduce food security emergencies in Africa. Johannesburg: NEPAD Planning and Coordinating Agency. 2013.

[67] World Meteorological Organisation [WMO]. State of the Climate in Africa 2021 (WMO-No. 1300). 2021. Accessed from: https://library.wmo.int/doc_num.php?explnum_id=11512 (February 21, 2023).

[68] Majdi F, Hosseini SA, Karbalaee A, Kaseri M, Marjanian S. Future projection of precipitation and temperature changes in the Middle East and North Africa (MENA) region based on CMIP6. Theoretical and Applied Climatology. 2022:1–14.

[69] Zizinga A, Mwanjalolo J-GM, Tietjen B, Bedadi B, Pathak H, Gabiri G, et al. Climate change and maize productivity in Uganda: Simulating the impacts and alleviation with climate smart agriculture practices. Agricultural Systems. 2022; 199:103407.

[70] Charnley GE, Kelman I, Murray KA. Drought-related cholera outbreaks in Africa and the implications for climate change: a narrative review. Pathogens and global health. 2022;116(1):3–12. doi: 10.1080/20477724.2021.1981716 34602024 PMC8812730

[71] Spinoni J, Naumann G, Carrao H, Barbosa P, Vogt J. World drought frequency, duration, and severity for 1951–2010. International Journal of Climatology. 2014;34(8):2792–804.

[72] Qu C, Hao X, Qu JJ. Monitoring extreme agricultural drought over the Horn of Africa (HOA) using remote sensing measurements. Remote Sensing. 2019;11(8):902.

[73] Otto FE, Wolski P, Lehner F, Tebaldi C, Van Oldenborgh GJ, Hogesteeger S, et al. Anthropogenic influence on the drivers of the Western Cape drought 2015–2017. Environmental Research Letters. 2018;13(12):124010.

[74] Sousa PM, Blamey RC, Reason CJ, Ramos AM, Trigo RM. The ‘Day Zero’Cape Town drought and the poleward migration of moisture corridors. Environmental Research Letters. 2018;13(12):124025.

[75] Ishumael S, Godwell N. An evaluation of climate change impacts on livelihoods of peasants in Makonde communal lands in Zimbabwe. Africa insight. 2015;44(4):90–105.

[76] Habtemariam LT, Gandorfer M, Kassa GA, Heissenhuber A. Factors influencing smallholder farmers’ climate change perceptions: a study from farmers in Ethiopia. Environmental management. 2016; 58:343–58. doi: 10.1007/s00267-016-0708-0 27179801

[77] Adeyeye SAO, Adebayo-Oyetoro AO, Tiamiyu HK. Poverty and malnutrition in Africa: a conceptual analysis. Nutrition & Food Science. 2017.

[78] Agyei-Mensah S, de-Graft Aikins A. Epidemiological transition and the double burden of disease in Accra, Ghana. Journal of urban health. 2010; 87:879–97. 20803094 10.1007/s11524-010-9492-y PMC2937133

[79] Mare F, Bahta YT, Van Niekerk W. The impact of drought on commercial livestock farmers in South Africa. Development in Practice. 2018;28(7):884–98.

[80] Perkins-Kirkpatrick S, Lewis S. Increasing trends in regional heatwaves. Nature communications. 2020;11(1):3357. doi: 10.1038/s41467-020-16970-7 32620857 PMC7334217

[81] Russo S, Sterl A. Global changes in indices describing moderate temperature extremes from the daily output of a climate model. Journal of Geophysical Research: Atmospheres. 2011;116(D3).

[82] Thornton PK, van de Steeg J, Notenbaert A, Herrero M. The impacts of climate change on livestock and livestock systems in developing countries: A review of what we know and what we need to know. Agricultural systems. 2009;101(3):113–27.

[83] Bezner Kerr R, Naess LO, Allen‐O’Neil B, Totin E, Nyantakyi‐Frimpong H, Risvoll C, et al. Interplays between changing biophysical and social dynamics under climate change: Implications for limits to sustainable adaptation in food systems. Global Change Biology. 2022;28(11):3580–604. doi: 10.1111/gcb.16124 35129261

[84] Olds P, Kachimanga C, Talama G, Mailosi B, Ndarama E, Totten J, et al. Non-communicable disease burden among inpatients at a rural district hospital in Malawi. Global Health Research and Policy. 2023;8(1):4. doi: 10.1186/s41256-023-00289-z 36810123 PMC9945353

[85] McMichael AJ, Lindgren E. Climate change: present and future risks to health, and necessary responses. Journal of internal medicine. 2011;270(5):401–13. doi: 10.1111/j.1365-2796.2011.02415.x 21682780

[86] Largeron Y, Guichard F, Roehrig R, Couvreux F, Barbier J. The April 2010 North African heatwave: when the water vapor greenhouse effect drives nighttime temperatures. Climate Dynamics. 2020; 54:3879–905.

[87] McEvoy D, Ahmed I, Mullett J. The impact of the 2009 heat wave on Melbourne’s critical infrastructure. Local environment. 2012;17(8):783–96.

[88] Rübbelke D, Vögele S. Impacts of climate change on European critical infrastructures: the case of the power sector. Environmental science & policy. 2011;14(1):53–63.

[89] Pasquini L, van Aardenne L, Godsmark CN, Lee J, Jack C. Emerging climate change-related public health challenges in Africa: A case study of the heat-health vulnerability of informal settlement residents in Dar es Salaam, Tanzania. Science of the total environment. 2020; 747:141355. doi: 10.1016/j.scitotenv.2020.141355 32777515

[90] Epstein Y, Yanovich R. Heatstroke. New England Journal of Medicine. 2019;380(25):2449–59. 31216400 10.1056/NEJMra1810762

[91] Kenny GP, Sigal RJ, McGinn R. Body temperature regulation in diabetes. Temperature. 2016;3(1):119–45. doi: 10.1080/23328940.2015.1131506 27227101 PMC4861190

[92] Adesina OS. Globalization, migration and the plight of Nigerians in South Africa. Nigeria-South Africa Relations and Regional Hegemonic Competence. 2019:109–27.

[93] Czaika M, De Haas H. The globalization of migration: Has the world become more migratory? International Migration Review. 2014;48(2):283–323.

[94] Galea S, Vlahov D. Urban health: evidence, challenges, and directions. Annu Rev Public Health. 2005; 26:341–65. doi: 10.1146/annurev.publhealth.26.021304.144708 15760293

[95] Omram AR. The epidemiologic transition: a theory of the epidemiology of population change. Bulletin of the World Health Organization. 2001;79(2):161–70. 11246833 PMC2566347

[96] Popkin BM. Global nutrition dynamics: the world is shifting rapidly toward a diet linked with noncommunicable diseases–. The American journal of clinical nutrition. 2006;84(2):289–98. 16895874 10.1093/ajcn/84.1.289

[97] Peltzer K, Pengpid S. Sociocultural factors and lifestyle behaviors associated with overweight and obesity among rural South African adolescents. International Journal of Environmental Research and Public Health. 2020;17(3):1–11.

[98] Chege CG, Andersson CI, Qaim M. Impacts of supermarkets on farm household nutrition in Kenya. World Development. 2015; 72:394–407.

[99] Timmer CP. Do supermarkets change the food policy agenda? World Development. 2009;37(11):1812–9.

[100] Stranges S, Luginaah I. Nutrition and health: Time for a paradigm shift for climate change. Nutrition, Metabolism and Cardiovascular Diseases. 2022;32(12):2782–5. 10.1016/j.numecd.2022.09.023 36333202

[101] Leroy F, Smith NW, Adesogan AT, Beal T, Iannotti L, Moughan PJ, et al. The role of meat in the human diet: evolutionary aspects and nutritional value. Animal Frontiers. 2023;13(2):11–8. 37073319 10.1093/af/vfac093 PMC10105836

[102] Giromini C, Givens DI. Benefits and Risks Associated with Meat Consumption during Key Life Processes and in Relation to the Risk of Chronic Diseases. Foods. 2022;11(14):2063. doi: 10.3390/foods11142063 35885304 PMC9318327

[103] Wolk A. Potential health hazards of eating red meat. Journal of internal medicine. 2017;281(2):106–22. doi: 10.1111/joim.12543 27597529

[104] Udayar SE, Kumar R, Kumar P, Vairamuthu S, Thatuku S. Study of cardiovascular risk factors among transport drivers in rural area of Andhra Pradesh. National Journal of Community Medicine. 2015;6(04):566–70.

[105] Lalla-Edward ST, Fischer AE, Venter WF, Scheuermaier K, Meel R, Hankins C, et al. Cross-sectional study of the health of southern African truck drivers. BMJ open. 2019;9(10): e032025. doi: 10.1136/bmjopen-2019-032025 31662399 PMC6830589

[106] Anderzén I, Arnetz BB. The impact of a prospective survey-based workplace intervention program on employee health, biologic stress markers, and organizational productivity. Journal of occupational and environmental medicine. 2005:671–82. doi: 10.1097/01.jom.0000167259.03247.1e 16010194

[107] Akande OW, Akande TM. COVID-19 pandemic: A global health burden. Niger Postgrad Med J. 2020;27(3):147–55. doi: 10.4103/npmj.npmj_157_20 32687112

[108] Edelstein M, Angelides P, Heymann DL. Ebola: the challenging road to recovery. The Lancet. 2015;385(9984):2234–5. doi: 10.1016/S0140-6736(15)60203-3 25678357

[109] Kangmennaang J, Siiba A, Dassah E, Kansanga M. The role of social support and the built environment on diabetes management among structurally exposed populations in three regions in Ghana. BMC Public Health. 2023;23(1):1–12. 38093227 10.1186/s12889-023-17376-y PMC10717308

