# Supplementary material for: The relationship between climate change, globalization and non-communicable diseases in Africa: A systematic review
Source: PLoS One. 2024 Feb 23;19(2):e0297393. doi: 10.1371/journal.pone.0297393 (PMC10889617; doi:10.1371/journal.pone.0297393)
Supplement: S1 Table — (DOCX) [file pone.0297393.s001.docx]

**S1 Table: Key Search Strategy**

| **DATABASE** | **NON-COMMUNICABLE DISEASES** | **CLIMATE CHANGE AND NCDS** | **GLOBALIZATION AND NCDS** | **ENVIRONMENTAL CHANGE AND NCDS** |
| --- | --- | --- | --- | --- |
| Web of Science | “Non-communicable diseases: OR “Non-Communicable Diseases” OR “NCDs” AND “Africa” | “Climate change” AND “Non-communicable diseases” OR “Non-communicable diseases” OR “NCDs” AND “Africa” | “Globalization” OR “Globalization” AND “Non-communicable diseases” OR “Non-communicable diseases” OR “NCDs” AND “Africa” | “Environmental Change” AND “Non-communicable diseases” OR “Non-communicable diseases” OR “NCDs” AND “Africa” |
| Scopus | ✓ | ✓ | ✓ | ✓ |
| Science Direct | ✓ | ✓ | ✓ | ✓ |
| PubMed | ✓ | ✓ | ✓ | ✓ |
| ProQuest | ✓ | ✓ | ✓ | ✓ |
| Medline (through Ovid) | ✓ | ✓ | ✓ | ✓ |
| Google Scholar | ✓ | ✓ | ✓ | ✓ |
| Global Health (through Ovid) | ✓ | ✓ | ✓ | ✓ |
