# Supplementary material for: The relationship between climate change, globalization and non-communicable diseases in Africa: A systematic review
Source: PLoS One. 2024 Feb 23;19(2):e0297393. doi: 10.1371/journal.pone.0297393 (PMC10889617; doi:10.1371/journal.pone.0297393)
Supplement: S2 Table — (DOCX) [file pone.0297393.s002.docx]

**S2 Table: PRISMA Checklist of Systematic Review**

| **Cross-sectional Studies** | **1** | **2** | **3** | **4** | **5** | **6** | **7** | **8** | **9** | **10** | **11** | **12** | **13** | **14** | **15** | **16** | **17** |
| --- | --- | --- | --- | --- | --- | --- | --- | --- | --- | --- | --- | --- | --- | --- | --- | --- | --- |
| Criteria for inclusion in the sample defined? | x | ✓ | ✓ | ✓ | ✓ | ✓ | ✓ | ✓ | ✓ | ✓ | ✓ | ✓ | ✓ | ✓ | ✓ | ✓ | ✓ |
| Study subjects and setting described in detail? | ✓ | ✓ | ✓ | ✓ | ✓ | ✓ | ✓ | ✓ | ✓ | ✓ | ✓ | ✓ | ✓ | ✓ | ✓ | ✓ | ✓ |
| Exposure measured in a valid and reliable way? | ✓ | ✓ | ✓ | ✓ | ✓ | ✓ | ✓ | ✓ | ✓ | ✓ | ✓ | ✓ | ✓ | ✓ | ✓ | ✓ | ✓ |
| Objective, standard criteria used for measurement of the condition? | ✓ | ✓ | ✓ | ✓ | ✓ | ✓ | ✓ | ✓ | ✓ | ✓ | ✓ | ✓ | ✓ | ✓ | ✓ | ✓ | ✓ |
| Confounding factors (CF) identified? | ✓ | ✓ | ✓ | x | ✓ | ✓ | ✓ | ✓ | ✓ | ✓ | ✓ | ✓ | x | x | n/a | ✓ | ✓ |
| Strategies to deal with CFs stated? | x | ✓ | ✓ | n/a | ✓ | ✓ | ✓ | ✓ | ✓ | ✓ | ✓ | ✓ | x | x | n/a | ✓ | ✓ |
| Outcomes measured in a valid and reliable way? | n/a | ✓ | ✓ | ✓ | ✓ | ✓ | ✓ | ✓ | ✓ | ✓ | ✓ | ✓ | ✓ | ✓ | ✓ | ✓ | ✓ |
| Appropriate statistical analysis used? | ✓ | ✓ | ✓ | ✓ | ✓ | ✓ | ✓ | ✓ | ✓ | ✓ | ✓ | ✓ | ✓ | ✓ | ✓ | ✓ | ✓ |
| **Qualitative Research** | | | | | | | | **18** | **19** | **20** | **21** | **22** | **23** | **24** | **25** | **26** | **27** |
| Is there congruity between the stated philosophical perspective and the research methodology? | | | | | | | | ✓ | ✓ | ✓ | ✓ | ✓ | ✓ | ✓ | ✓ | ✓ | ✓ |
| Is there congruity between the research methodology and the research question or objectives? | | | | | | | | ✓ | ✓ | ✓ | ✓ | ✓ | ✓ | ✓ | ✓ | ✓ | ✓ |
| Is there congruity between the research methodology and the methods used to collect data? | | | | | | | | n/a | n/a | ✓ | ✓ | ✓ | ✓ | ✓ | ✓ | ✓ | n/a |
| Is there congruity between the research methodology and the representation and analysis of data? | | | | | | | | ✓ | ✓ | ✓ | ✓ | ✓ | ✓ | ✓ | ✓ | ✓ | ✓ |
| Is there congruity between the research methodology and the interpretation of results? | | | | | | | | ✓ | ✓ | ✓ | ✓ | ✓ | ✓ | ✓ | ✓ | ✓ | ✓ |
| Is there a statement locating the researcher culturally or theoretically? | | | | | | | | ✓ | ✓ | ✓ | ✓ | ✓ | ✓ | ✓ | ✓ | ✓ | ✓ |
| Is the influence of the researcher on the research, and vice- versa, addressed? | | | | | | | | n/a | n/a | n/a | n/a | ✓ | n/a | n/a | ✓ | n/a | n/a |
| Are participants, and their voices, adequately represented? | | | | | | | | n/a | n/a | n/a | n/a | ✓ | n/a | n/a | ✓ | n/a | n/a |
| Is the research ethical according to current criteria or, for recent studies, and is there evidence of ethical approval by an appropriate body? | | | | | | | | n/a | n/a | n/a | n/a | n/a | n/a | n/a | ✓ | n/a | n/a |
| Do the conclusions drawn in the research report flow from the analysis, or interpretation, of the data? | | | | | | | | ✓ | ✓ | ✓ | ✓ | ✓ | ✓ | ✓ | ✓ | ✓ | ✓ |

Modified from: Joanna Briggs Institute (JBI) (2023) (https://synthesismanual.jbi.global).

**Notes for citations:** 1 = [47], 2 = [57], 3 = [19], 4 = [42], 5 = [52], 6 = [20], 7 = [38], 8 = [39], 9 = [43], 10 = [45], 11 = [46], 12 = [49], 13 = [28], 14 = [50], 15 = [15], 16 = [51], 17 = [40], 18 = [53], 19 = [54], 20 = [55], 21 = [44], 22 = [48], 23 = [59], 24 = [27], 25 = [37], 26, [60], 27 = [56], n/a = not applicable).
